# Associations between Adipokines and Metabolic Dysfunction-Associated Fatty Liver Disease Using Three Different Diagnostic Criteria

**DOI:** 10.3390/jcm12062126

**Published:** 2023-03-08

**Authors:** Jie Pan, Yijie Ding, Yan Sun, Qiuyan Li, Tianyi Wei, Yingying Gu, Yujia Zhou, Nengzhi Pang, Lei Pei, Sixi Ma, Mengqi Gao, Ying Xiao, De Hu, Feilong Wu, Lili Yang

**Affiliations:** 1 Department of Nutrition, Guangdong Provincial Key Laboratory of Food, Nutrition and Health, School of Public Health, Sun Yat-sen University, Guangzhou 510080, China; 2 Department of Obstetrics, The First Women and Children’s Hospital of Huizhou, Huizhou 516000, China

**Keywords:** metabolic dysfunction-associated fatty liver disease, MAFLD, adipokines, adiponectin, adipsin, visfatin, diagnostic criteria

## Abstract

Background: A panel of experts proposed a new definition of metabolic dysfunction-associated fatty liver disease (MAFLD) in 2020. To date, the associations between adipokines, such as adiponectin, adipsin, and visfatin and MAFLD remain unclear. Therefore, we aimed to evaluate the associations between each of these three adipokines and MAFLD using different diagnostic criteria. Methods: In total, 221 participants were included in our study based on medical examination. Detailed questionnaire information, physical examination, abdominal ultrasound, and blood-biochemical-test indexes were collected. The levels of adipokines were tested by using an enzyme immunoassay. Logistic regression models were used to assess the associations of the adipokines with MAFLD. Results: In total, 122 of the participants were diagnosed with MAFLD. Higher levels of adipsin and lower levels of adiponectin were found in the MAFLD group than in the non-MAFLD group (all *p* < 0.05). According to the logistic regression analysis, the ORs were 0.11 (95% CI: 0.05–0.23) for adiponectin, 4.46 (95% CI: 2.19–9.12) for adipsin, and 0.51 (95% CI: 0.27–0.99) for visfatin when comparing the highest tertile with the lowest tertile (all *p*-trend < 0.05). The inverse association between adiponectin and MAFLD was strongest when T2DM was used as the diagnostic criterion alone, and the positive association between adipsin and MAFLD was strongest when BMI was used as the diagnostic criterion alone. There was no significant association between visfatin and MAFLD, regardless of whether each of BMI, T2DM, or metabolic dysregulation (MD) was used as the diagnostic criterion for MAFLD alone. Conclusion: Adipsin levels were positively associated with MAFLD and adiponectin levels were inversely associated with MAFLD. The strength of these associations varied according to the different diagnostic criteria for MAFLD.

## 1. Introduction

The term “nonalcoholic fatty-liver disease” (NAFLD) was first coined by Ludwig et al. in 1980 to describe the disease of fatty liver without significant alcohol consumption [1]. In general, NAFLD is defined as steatosis in more than 5% of hepatocytes without significant alcohol consumption and other known causes of liver disease [2,3,4,5]. Nonalcoholic fatty-liver disease is related to metabolic comorbidities [6,7], and the role of metabolic risk factors in the progression of NAFLD should not be ignored. The pathogenesis of NAFLD is complex. Genetic susceptibility, environment, and metabolic risk factors interact with each other, which promote the development of NAFLD and increase the risk of disease progression [8]. The nomenclature and diagnostic criteria of NAFLD were not revisited until 2020.

A panel of international experts proposed a new definition of metabolic dysfunction-associated fatty liver disease (MAFLD) in 2020 [9]. Metabolic dysfunction-associated fatty liver disease is a hepatic manifestation of systemic metabolic dysfunction [8]. It is diagnosed based on hepatic steatosis and one of three other criteria: overweight/obesity, type 2 diabetes mellitus, or metabolic dysregulation [9]. The overall prevalence of MAFLD was 38.77% (95% CI: 32.94–44.95%) according to a meta-analysis [10], notably exceeding previous estimates of the global prevalence of NAFLD [11,12,13].

The factors secreted by adipose tissue are collectively referred to as adipokines [14]. Adipokines signal key organs to maintain metabolic homeostasis, and their dysfunction has been implicated in a vast range of metabolic disorders [15]. Adiponectin is the most abundant peptide secreted by adipocytes [16] and has anti-inflammatory properties in the liver [17]. The adiponectin levels were low in patients with fatty-liver disease [18]. The addition of adiponectin in mice can significantly improve hepatomegaly and steatosis [19]. Adipsin (also known as complement factor D) was identified as the first adipokine to be highly expressed in adipocytes [20,21]. It plays a key role in glycolipid metabolism, energy balance, and maintaining islet beta cell function [22,23,24]. Visfatin is an adipokine produced and secreted primarily by visceral white adipose tissue [25]. According to a literature review, controversy remains in current studies on the role of visfatin in insulin resistance, hepatic steatosis, and hepatic fibrosis [26].

Associations between MAFLD and the three adipokines, adiponectin, adipsin, and visfatin, have not been reported until now. In this study, we aimed to investigate the associations of adiponectin, adipsin, and visfatin with MAFLD by using different diagnostic criteria. We also analyzed the differences in adipokine levels between participants with and without metabolic dysregulation.

## 2. Materials and Methods

### 2.1. Study Population

**Figure 1:**
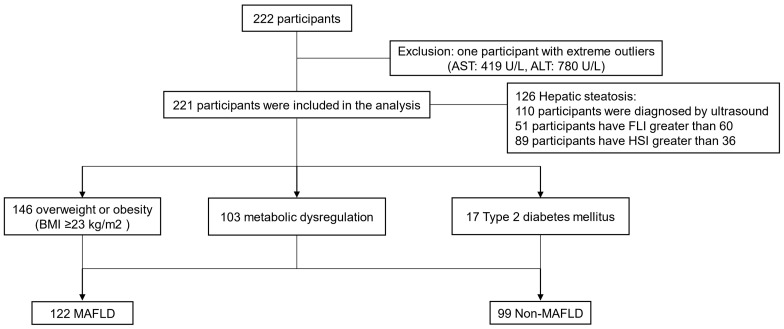
Specific grouping characteristics of the research subjects in this study.

In total, 221 participants were included in our study based on medical examination at the Physical Examination Center of the Third Affiliated Hospital of Sun Yat-sen University from April 2016 to September 2016. The grouping characteristics of the participants are presented in Figure 1.

This study was conducted according to the Declaration of Helsinki and was approved by the Ethics Committee of the School of Public Health at Sun Yat-sen University. Written informed consent was obtained from all participants.

### 2.2. Clinical- and Laboratory-Data Collection

Participants were interviewed face-to-face by trained investigators using structured questionnaires to collect information concerning demographic sociological characteristics, behavior, and lifestyle. A vertical ruler and digital scale were used to measure participants’ heights and weights, while an anthropometric tape was used to gauge waist circumference and hip circumference. Participants’ blood pressure was measured with an electronic sphygmomanometer on their left arm. Note that all the above measurements were conducted twice and the values were averaged. Body mass index (BMI) was calculated as weight (kg)/[height (m)]^2^.

The imaging of a normal liver can be identified by similarity in sonogram echoes between the kidney or spleen and liver. The following characteristics were used to detect fatty liver by ultrasonography: liver parenchyma appeared more echogenic than renal cortex, posterior-beam attenuation was present, vessels were less visible, and diaphragmatic appearance appeared abnormal [27,28].

A 10-milliliter fasting blood sample was obtained from each participant in the morning after overnight fasting (free of food intake for more than 8 h before blood drawing). The serum was separated into several aliquots and stored at −80 °C within 2 h. Blood biochemistry was examined and routine blood tests were performed in the clinical laboratory of the hospital. Serum alanine aminotransferase (ALT), aspartate aminotransferase (AST), gamma-glutamyl transferase (GGT), total cholesterol (TC), triglycerides (TG), low-density-lipoprotein cholesterol (LDL-C), high-density-lipoprotein cholesterol (HDL-C), uric acid (UA), and fasting glucose were measured by an automatic biochemical analyzer (Hitachi 7600, Tokyo, Japan). A quantitative sandwich enzyme-linked immunosorbent assay was used to test adiponectin and adipsin levels (R&D Systems, Minneapolis, MN, USA). A competitive enzyme immunoassay (RayBiotech, Norcross, GA, USA) was used to test visfatin levels.

### 2.3. Working Definitions

Hepatic steatosis can be diagnosed with abdominal ultrasound diagnosis or by fatty-liver index (FLI) greater than 60 [29] or hepatic-steatosis index (HSI) greater than 36 [30].

Metabolic-dysfunction-associated fatty-liver disease is diagnosed by hepatic steatosis (detected by imaging techniques, blood biomarkers/scores, or liver histology) combined with any one of the following three criteria: overweight/obesity, type 2 diabetes mellitus, or metabolic dysregulation. [9]. Overweight/obesity is defined as BMI ≥ 23 kg/m^2^ in Asians. An individual diagnosed with metabolic dysregulation (MD) has at least two metabolic risk abnormalities: (i) waist circumference ≥ 90/80 cm in Asian men and women; (ii) blood pressure ≥ 130/85 mmHg or specific drug treatment; (iii) plasma triglycerides (TG) ≥ 1.70 mmol/L or specific drug treatment; (iv) plasma HDL-C < 1.0 mmol/L for men and <1.3 mmol/L for women or specific drug treatment; (v) prediabetes (fasting glucose levels 5.6 to 6.9 mmol/L); (vi) homeostasis model assessment of insulin-resistance (HOMA-IR) score ≥ 2.5; and (vii) plasma high-sensitivity C-reactive protein (hs-CRP) level > 2 mg/L [9].

Hypertension was diagnosed by systolic blood pressure ≥140 mmHg or diastolic blood pressure ≥ 90 mmHg. Type 2 diabetes mellitus (T2DM) was defined by having fasting glucose ≥ 7.0 mmol/L or a history of T2DM. The definition of dyslipidemia was as follows: TC ≥ 6.2 mmol/L or TG ≥ 2.3 mmol/L or HDL-C < 1.0 mmol/L or LDL-C ≥ 4.1 mmol/L [31]. For the current study, smoking was defined as more than one cigarette per day for six months and alcohol drinking was defined as drinking at least one time per week for six months.

The FLI was calculated using the following formula [29]:

FLI = (e 0.953 × loge (triglycerides) + 0.139 × BMI + 0.718 × loge (ggt) + 0.053 × waist circumference − 15.745)/(1 + e 0.953 × loge (triglycerides) + 0.139 × BMI + 0.718 × loge (ggt) + 0.053 × waist circumference − 15.745) × 100

Triglycerides, mg/dL (1 mg/dL = 0.0113 mmol/L); BMI, kg/m^2^; GGT, U/L; waist circumference, cm.

The HSI was calculated using the following formula [30]:

HSI = 8 × ALT/AST ratio + BMI (+2, if DM; +2, if female)

### 2.4. Statistical Analysis

Continuous variables were presented as means ± standard deviation or median (interquartile range), depending on whether the data were normally distributed. Categorical variables were described as frequency (percentage). Independent-samples *t*-test, Mann–Whitney *U*-test, and Pearson chi-square test were used for comparison between two groups. Kruskal–Wallis test was used to compare multiple groups. The logistic regression model was used to examine the associations of adipokines with MAFLD. The lowest tertile of adipokine levels served as the reference group. Tests for trends across the tertile of adipokines levels were evaluated by using a median value within each tertile as a continuous value.

One participant had an AST level of 419 U/L and an ALT level of 780 U/L, which were considered as extreme outliers; he was therefore excluded. Eventually, 221 participants were included in the analysis. All statistical analyses were performed using SPSS 25.0 software (SPSS Inc., Chicago, IL, USA) and R version 4.2.0. The significance threshold was 0.05, and all tests were 2-sided.

## 3. Results

### 3.1. Comparison of Adipokines between the Non-MAFLD and MAFLD Group (Three Diagnostic Criteria)

**Figure 2:**
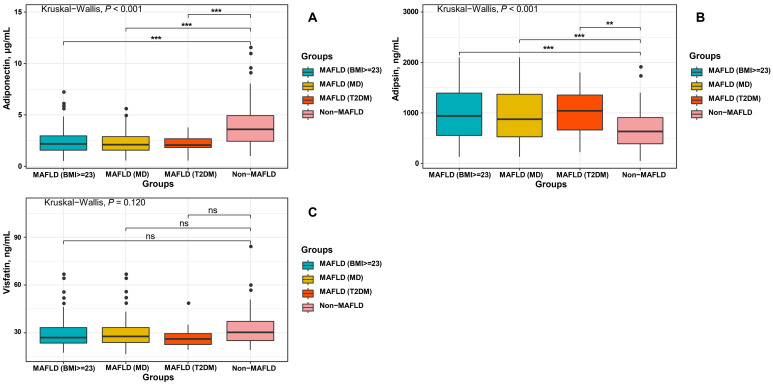
Comparison of adipokine levels ((**A**) adiponectin; (**B**) adipsin; (**C**) visfatin) between the non-MAFLD group and MAFLD group with different diagnostic criteria. In total, 114 cases of MAFLD were diagnosed by BMI, 87 by diagnosed by MD, and 15 MAFLD diagnosed by T2DM; 99 patients did not have MAFLD. MD: metabolic dysregulation. ns, *p* > 0.05; ** *p* < 0.01; *** *p* < 0.001.

As shown in Figure 2, higher levels of adipsin and lower levels of adiponectin were found in the MAFLD groups (regardless of whether the diagnostic criteria were BMI, MD, or T2DM) than in the non-MAFLD group (all *p* < 0.05). However, no statistical significance was found in the difference in visfatin levels between the non-MAFLD group and the MAFLD group (regardless of whether the diagnostic criteria were BMI, MD, or T2DM).

### 3.2. Comparison between the Non-MAFLD Group and the MAFLD Group

All the participants were divided into two groups (the non-MAFLD group and the MAFLD group) based on whether they had MAFLD. Through univariate comparisons between the two groups, the WC, WHR, BMI, SBP, DBP, TC, TG, LDL-C, fasting glucose, ALT, AST, GGT, UA, PLT, FLI score, and HSI score were found to be higher in the MAFLD group than in the non-MAFLD group. In addition, the MAFLD group contained a higher proportion of subjects who also had T2DM, hypertension, and dyslipidemia. It was noteworthy that the adipsin levels were significantly higher in the MAFLD group compared to those in the non-MAFLD group (889.06 (528.99–1379.60) ng/mL vs. 632.63 (379.32–908.19) ng/mL, *p* < 0.001). Conversely, those in the MAFLD group had lower levels of adiponectin in comparison with those in the non-MAFLD group, with levels of 2.17 (1.56–2.98) µg/mL and 3.60 (2.41–4.96) µg/mL, respectively (*p* < 0.001). The visfatin levels were similar between the two groups, with levels of 27.12 (23.49–33.33) ng/mL and 30.31 (25.08–37.46) ng/mL in the MAFLD and non-MAFLD groups, respectively (*p* = 0.052; Table 1).

### 3.3. Differences in the Distribution of Adipokine Tertile Levels between the MAFLD and Non-MAFLD Groups

**Figure 3:**
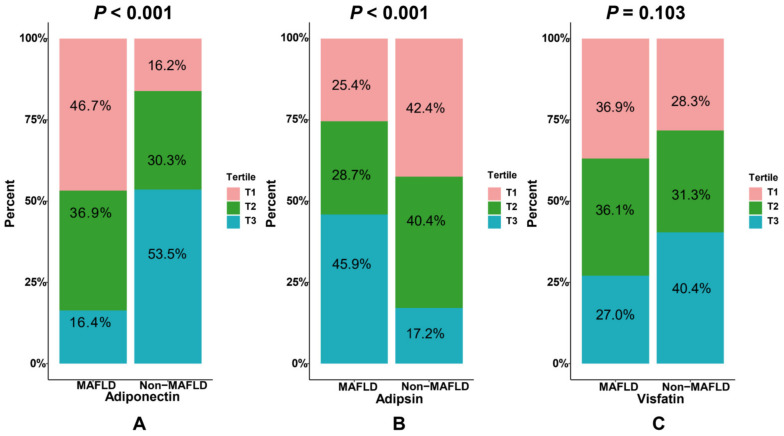
Distribution of adipokine tertile levels ((**A**) adiponectin; (**B**) adipsin; (**C**) visfatin) between the MAFLD and non-MAFLD groups.

Figure 3 shows the differences in the distribution of the adiponectin, adipsin, and visfatin tertile levels between the MAFLD and non-MAFLD groups. The highest tertile level (T3) of adiponectin accounted for more than 50% in the non-MAFLD group but less than 20% in the MAFLD group (*p* < 0.001). Conversely, the highest tertile (T3) of adipsin was 45.9% in the MAFLD group, but less than 20% in the non-MAFLD group (*p* < 0.001). The distribution of the visfatin levels did not differ significantly between the two groups (*p* = 0.103).

### 3.4. Associations of Adiponectin, Adipsin, and Visfatin with MAFLD

The ORs for the associations of MAFLD with adiponectin, adipsin, and visfatin are shown in Table 2. In model 1, the ORs of MAFLD were 0.11 (95% CI: 0.05–0.23; *p*-trend < 0.001) for adiponectin, 4.46 (95% CI: 2.19–9.12; *p*-trend < 0.001) for adipsin, and 0.51 (95% CI: 0.27–0.99; *p*-trend = 0.037) for visfatin in the highest tertile compared with those in the lowest tertile. In model 2, after adjusting for age, gender, smoking, drinking, physical activity, AST, and ALT, the ORs of MAFLD were 0.12 (95% CI: 0.05–0.30; *p*-trend < 0.001) for adiponectin, 6.90 (95% CI: 2.71–17.61; *p*-trend < 0.001) for adipsin, and 0.54 (95% CI: 0.24–1.20; *p*-trend = 0.122) for visfatin in the highest tertile compared with those in the lowest tertile.

**Figure 4:**
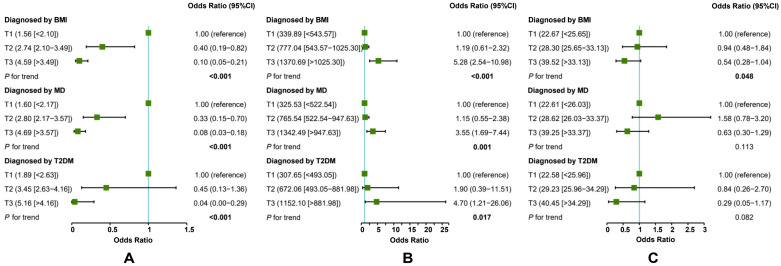
Crude models: associations of adiponectin (**A**), adipsin (**B**), and visfatin (**C**) with MAFLD. In total, 114 cases of MAFLD were diagnosed by BMI, 87 by MD, and 15 by T2DM. Control: 99 non-MAFLD. MD: metabolic dysregulation. Firth’s bias-reduced-penalized-likelihood logistic regression (diagnosed by T2DM).

**Figure 5:**
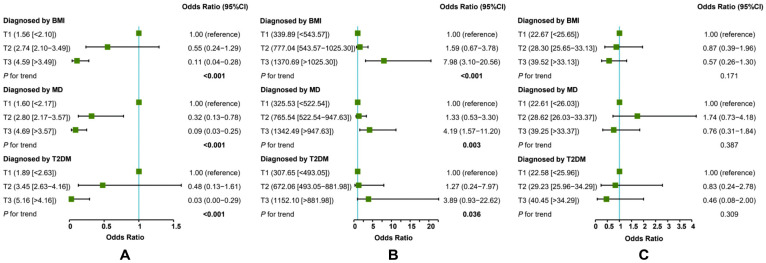
Adjusted models: associations of adiponectin (**A**), adipsin (**B**), and visfatin (**C**) with MAFLD. Adjusted for age, gender, smoking, drinking, physical activity, AST, and ALT (diagnosed by BMI and diagnosed by MD); adjusted for age, gender, smoking, drinking, and physical activity (diagnosed by T2DM). In total, 114 cases of MAFLD were diagnosed by BMI, 87 by MD, and 15 diagnosed by T2DM. Control: 99 non-MAFLD. MD: metabolic dysregulation. Firth’s bias-reduced-penalized-likelihood logistic regression (diagnosed by T2DM).

The inverse association between adiponectin and MAFLD (the highest tertile vs. the lowest tertile) was the strongest when T2DM was used as the diagnostic criterion alone (Figure 4A), and the positive association between adipsin and MAFLD (the highest tertile vs. the lowest tertile) was the strongest when BMI was used as the diagnostic criterion alone (Figure 4B). In addition, there were no significant associations between visfatin and MAFLD, regardless of whether each of BMI, MD, or T2DM was used as the diagnostic criterion alone for MAFLD (Figure 4C). After adjusting for multiple potential confounders, there were no significant changes in the associations between the three adipokines and MAFLD under the three diagnostic criteria (Figure 5).

### 3.5. Differences in Adipokine Levels in the Presence or Absence of Metabolic Dysregulation

**Figure 6:**
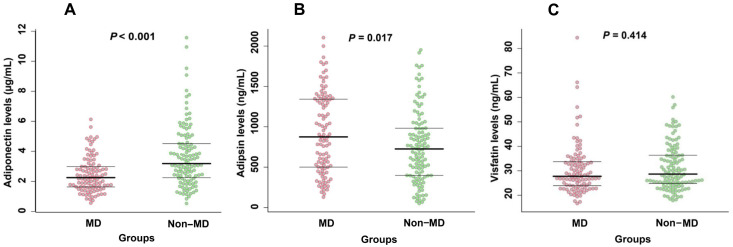
Differences in adipokine levels ((**A**) adiponectin; (**B**) adipsin; (**C**) visfatin) between the MD and non-MD groups. MD: metabolic dysregulation.

Compared with the group without MD, the group with MD had lower adiponectin levels (*p* < 0.001). Conversely, the MD group had higher adipsin levels than the non-MD group (*p* = 0.017). However, the visfatin levels did not differ significantly between the two groups and were similarly distributed (*p* = 0.414; Figure 6).

## 4. Discussion

According to the analysis of the 221 participants, we found higher levels of adipsin and lower levels of adiponectin in the MAFLD group than in the non-MAFLD group. The inverse association between adiponectin and MAFLD was at its strongest when T2DM was used as the diagnostic criterion alone, and the positive association between adipsin and MAFLD was the strongest when BMI was used as the diagnostic criterion alone. There was no significant association between visfatin and MAFLD, regardless of whether BMI, MD, or T2DM was used as the diagnostic criterion alone for MAFLD.

Adiponectin ameliorated hepatomegaly and steatosis and decreased the levels of liver enzymes in mice [19]. In a model of rhesus monkeys that spontaneously developed obesity and subsequently developed type 2 diabetes, the plasma adiponectin levels were also decreased [32]. A systematic review and meta-analysis also reported that higher adiponectin levels were consistently associated with a lower risk of type 2 diabetes in prospective studies of diverse populations [33]. Chow et al. reported an independent association between hypoadiponectinemia and hypertension [34]. Additionally, several studies reported lower adiponectin levels in patients with hepatic steatosis than in controls [35,36,37]. The research results regarding adiponectin in animals and humans are consistent: it appears to have a protective effect on metabolism-related diseases. Similarly, in our study, we found that the participants without MAFLD or MD had higher adiponectin levels compared with the controls and that adiponectin levels were inversely associated with MAFLD. This association was at its strongest when T2DM was used as the diagnostic criterion alone. After adjusting for potential confounding factors, the degree of this association was retained. This may have been due to the close association between adiponectin and T2DM. Currently, adiponectin is among the strongest and most consistent biochemical predictors of T2DM [38].

Adipsin increases lipid accumulation and adipocyte differentiation [39]. According to previous reports, in human studies of metabolism-related diseases (such as obesity, diabetes, and metabolic syndrome), adipsin levels were higher compared to controls [40,41,42,43]. Since the definition of MAFLD involves hepatic steatosis, overweight/obesity, metabolic dysregulation, and diabetes, it may be reasonable to speculate that increased circulating levels of adipsin are associated with MAFLD. In our study, we found that the people with MAFLD or MD had higher levels of adipsin than the controls and that adipsin levels were positively associated with MAFLD. This association was at its strongest when BMI was used as the diagnostic criterion alone, and this phenomenon persisted after adjusting for several potential confounding factors. On one hand, adipsin may be more strongly associated with BMI than T2DM or MD. On the other, the association may have arisen because the sample size of MAFLD diagnosed by BMI alone was the largest.

With respect to visfatin, it has been reported that the serum visfatin concentration was significantly higher in obese women compared to non-obese women [44]. A systematic review and meta-analysis found that plasma visfatin was significantly increased in subjects diagnosed with overweight/obesity, T2DM, and metabolic syndrome [45]. However, another review indicated that visfatin levels were not associated with NAFLD, the presence or severity of hepatic steatosis, NASH, or gender [46]. Ismaiel et al. reported that no significant difference in serum visfatin levels in MAFLD patients compared with controls was found [16]. Currently, the findings regarding the association of visfatin with metabolism-related diseases or liver diseases are still controversial. In this study, we found no difference in visfatin levels between the MAFLD and non-MAFLD groups and the distribution of visfatin levels was similar between the MD and non-MD groups. In addition, no association was observed between visfatin and MAFLD, regardless of whether each of BMI, MD, or T2DM was used as the diagnostic criterion alone. There are three possible explanations for the results of this study: (1) the association between visfatin itself and metabolism-related diseases is relatively weak; (2) different population sources and study designs can influence results; and (3) the sample size of this study was not sufficiently large.

The new definition of MAFLD may reduce patients’ confusion about the true cause of fatty-liver disease, which, in turn, could lead to better communication between doctors and patients. Using “positive” features to diagnose MAFLD can better stratify the risks of patients, and, subsequently, help to take targeted prevention and treatment measures to improve clinical efficacy. This is difficult to accomplish with NAFLD, which is defined by using certain conditions as the exclusion criteria. Unlike cases of NAFLD only, the MAFLD criteria can help identify a meaningful group of people with more comorbidities and worse prognoses [47]. Lin et al. reported that the MAFLD definition is more practical for identifying patients with fatty-liver disease with a high risk of disease progression [48].

This change in terminology will affect drug development and biomarker discovery. The use of different diagnostic criteria may influence the potential biomarkers of MAFLD. Although epidemiologic associations between adipokines and metabolism-related diseases have been established, the causal relationship and the exact molecular mechanisms are still unclear. Large-sample prospective-study designs are needed to further validate whether these adipokines can act as potential biomarkers of MAFLD under different diagnostic conditions.

Ultrasound is less sensitive for detecting hepatic steatosis in individuals with steatosis of less than 20% or with a BMI greater than 40 [9]. Therefore, in our study, we diagnosed hepatic steatosis by ultrasound or fatty-liver index or hepatic-steatosis index, which is a more reliable approach than using ultrasound alone. In addition, FLI and HSI have higher cut-off values for diagnosing hepatic steatosis (60 and 36, respectively), so there is less misclassification bias.

Nevertheless, there are some limitations in our study. First, this is a descriptive study, which can only draw epidemiological associations, not causal inferences, although it can provide clues for further mechanistic studies. Second, the effect of HOMA-IR and hs-CRP on MAFLD could not be investigated due to the lack of data on their levels. Third, the participants in this study were mainly men, so the conclusions drawn from the study should be extended to women with caution.

In conclusion, our study found that the MAFLD group had higher levels of adipsin and lower levels of adiponectin than the non-MAFLD group (regardless of whether each of BMI, MD, or T2DM was used as the diagnostic criterion alone for MAFLD). The adipsin levels were positively associated with MAFLD, the adiponectin levels were inversely associated with MAFLD, and the strength of these associations varied according to different diagnostic criteria. Furthermore, the difference in visfatin levels between the MAFLD and non-MAFLD groups and its association with MAFLD were not significant.

## Figures and Tables

**Table 1:** Clinical characteristics of participants in the non-MAFLD and MAFLD groups.

Variables	Non-MAFLD (*n* = 99)	MAFLD (*n* = 122)	*p* Value
Age, years	32.00 (28.00–37.00)	33.00 (29.00–36.00)	0.306
Male, *n* (%)	80 (80.81)	101 (82.79)	0.704
Smoking, *n* (%)	14 (14.14)	17 (13.93)	0.965
Drinking, *n* (%)	24 (24.24)	26 (21.31)	0.605
Physical activity, h/week	5.00 (3.00–9.50)	4.08 (2.38–8.00)	0.100
WC, cm	80.29 ± 6.63	93.30 ± 7.66	<0.001
WHR	0.85 ± 0.06	0.91 ± 0.05	<0.001
BMI, kg/m^2^	22.09 (20.76–23.67)	25.97 (24.60–27.74)	<0.001
Adipsin, ng/mL	632.63 (379.32–908.19)	889.06 (528.99–1379.60)	<0.001
Visfatin, ng/mL	30.31 (25.08–37.46)	27.12 (23.49–33.33)	0.052
Adiponectin, µg/mL	3.60 (2.41–4.96)	2.17 (1.56–2.98)	<0.001
SBP, mmHg	116.99 ± 12.31	126.46 ± 13.92	<0.001
DBP, mmHg	72.65 ± 8.16	80.19 ± 8.78	<0.001
TC, mmol/L	4.74 ± 0.71	5.05 ± 0.79	0.003
TG, mmol/L	1.03 (0.81–1.30)	1.73 (1.31–2.42)	<0.001
LDL-C, mmol/L	3.00 ± 0.69	3.25 ± 0.69	0.009
HDL-C, mmol/L	1.35 ± 0.30	1.18 ± 0.23	<0.001
Fasting glucose, mmol/L	5.01 (4.66–5.29)	5.09 (4.77–5.42)	0.044
ALT, U/L	18.00 (14.00–23.00)	35.00 (23.00–60.00)	<0.001
AST, U/L	19.00 (17.00–24.00)	25.50 (20.00–31.00)	<0.001
AST/ALT	1.11 (0.86–1.33)	0.71 (0.54–0.89)	<0.001
GGT, U/L	19.00 (15.00–26.00)	37.00 (24.00–53.50)	<0.001
UA, µmol/L	393.12 ± 85.84	473.41 ± 109.65	<0.001
Albumin, g/dl	4.82 ± 0.23	4.84 ± 0.23	0.578
PLT, 10^9^/L	242.00 ± 42.80	263.11 ± 54.86	0.010
Hypertension, *n* (%)	4 (4.04)	28 (22.95)	<0.001
Dyslipidemia, *n* (%)	21 (21.21)	55 (45.08)	<0.001
T2DM, *n* (%)	2 (2.02)	15 (12.30)	0.004
FLI	13.98 (6.06–23.81)	51.73 (37.10–74.43)	<0.001
HSI	30.26 ± 3.63	39.04 ± 5.25	<0.001

Abbreviations: WC, waist circumference; WHR, waist-to-hip ratio; BMI, body mass index; SBP, systolic blood pressure; DBP, diastolic blood pressure; TC, total cholesterol; TG, triglyceride; LDL-C, low-density-lipoprotein cholesterol; HDL-C, high-density-lipoprotein cholesterol; ALT, alanine aminotransferase; AST, aspartate aminotransferase; GGT, gamma-glutamyltransferase; UA, uric acid; PLT, platelet count; T2DM, type 2 diabetes mellitus; FLI, fatty-liver index; HSI, hepatic steatosis index.

**Table 2:** Associations of adiponectin, adipsin, and visfatin with MAFLD.

	Model 1		Model 2	
	OR (95% CI)	*p* Value	OR (95% CI)	*p* Value
Adiponectin (µg/mL, median (range))				
T1 (1.55 (<2.09))	Reference		Reference	
T2 (2.71 (2.09–3.48))	0.42 (0.21–0.87)	0.019	0.55 (0.24–1.28)	0.165
T3 (4.59 (>3.48))	0.11 (0.05–0.23)	<0.001	0.12 (0.05–0.30)	<0.001
*p* for trend	<0.001		<0.001	
Adipsin (ng/mL, median (range))				
T1 (339.89 (<540.67))	Reference		Reference	
T2 (772.11 (540.67–1008.49))	1.19 (0.62–2.27)	0.607	1.52 (0.66–3.52)	0.327
T3 (1366.98 (>1008.49))	4.46 (2.19–9.12)	<0.001	6.90 (2.71–17.61)	<0.001
*p* for trend	<0.001		<0.001	
Visfatin (ng/mL, median (range))				
T1 (22.63 (<25.62))	Reference		Reference	
T2 (28.30 (25.62–3.02))	0.88 (0.46–1.71)	0.712	0.84 (0.38–1.86)	0.668
T3 (39.34 (>33.02))	0.51 (0.27–0.99)	0.048	0.54 (0.24–1.20)	0.130
*p* for trend	0.037		0.122	

Notes: 122 MAFLD; 99 non-MAFLD. Model 1: crude model; Model 2: adjusted for age, gender, smoking, drinking, physical activity, AST, and ALT.

## Data Availability

The datasets generated during and/or analyzed during the current study are available from the corresponding author upon reasonable request.
